# Exploring Research on the Construction and Application of Knowledge Graphs for Aircraft Fault Diagnosis

**DOI:** 10.3390/s23115295

**Published:** 2023-06-02

**Authors:** Xilang Tang, Guo Chi, Lijie Cui, Andrew W. H. Ip, Kai Leung Yung, Xiaoyue Xie

**Affiliations:** 1 Equipment Management and Unmanned Aerial Vehicle Engineering School, Air Force Engineering University, Xi’an 710051, China; tangxilang@sina.com (X.T.);; 2 College of Equipment Management and Support, Engineering University of PAP, Xi’an 710086, China; 3 Department of Industrial and Systems Engineering, The Hong Kong Polytechnic University, Hong Kong, China

**Keywords:** aircraft fault diagnosis, knowledge graph, deep learning, fault knowledge extraction, question-answering system

## Abstract

Fault diagnosis is crucial for repairing aircraft and ensuring their proper functioning. However, with the higher complexity of aircraft, some traditional diagnosis methods that rely on experience are becoming less effective. Therefore, this paper explores the construction and application of an aircraft fault knowledge graph to improve the efficiency of fault diagnosis for maintenance engineers. Firstly, this paper analyzes the knowledge elements required for aircraft fault diagnosis, and defines a schema layer of a fault knowledge graph. Secondly, with deep learning as the main method and heuristic rules as the auxiliary method, fault knowledge is extracted from structured and unstructured fault data, and a fault knowledge graph for a certain type of craft is constructed. Finally, a fault question-answering system based on a fault knowledge graph was developed, which can accurately answer questions from maintenance engineers. The practical implementation of our proposed methodology highlights how knowledge graphs provide an effective means of managing aircraft fault knowledge, ultimately assisting engineers in identifying fault roots accurately and quickly.

## 1. Introduction

Aircraft fault diagnosis is the process of identifying the root cause of system function failures in aircraft. The ability to locate the faulty component quickly and accurately is crucial for repairing aircraft and ensuring their proper functioning. Recently, the advances in aircraft health management technology have enabled certain faults that are predicted during the design phase to be identified by the aircraft itself [1]. Despite these advancements, a significant number of faults still rely on the expertise of engineers when they cannot be predicted during the design phase. Given the complexity of aircraft, fault diagnosis remains a knowledge-intensive activity [2,3], where maintenance engineers need to spend substantial amounts of time reading related literature materials and mastering a knowledge of associated faults, such as in aircraft structure, function, and signals, as well as possible fault causes. Armed with this knowledge, engineers conduct observations, measurements, and other test methods to progressively locate the faults. Unfortunately, this diagnostic process is frequently inefficient and can lead to prolonged aircraft downtime, culminating in severe economic losses.

Throughout the life cycle of aircraft, a significant number of documents related to failures are generated [4], including FMECA (Failure Mode, Effect, and Criticality Analysis) documents, aircraft maintenance manuals, diagnostic records, and fault analysis reports, among others. These resources provide valuable knowledge for the diagnosis of aircraft faults [5,6]. However, due to the heterogeneity of the data, these resources exist as fragmented “information islands” [7], hindering effective knowledge sharing and leading to inefficient resource utilization. Therefore, in order to achieve comprehensive fault knowledge sharing, it is important to integrate industrial information [8]. New artificial intelligence methods need to be developed to fully harness this vast textual data of failures, extract and share fault knowledge, and create an “intelligent diagnostic expert by one’s side” for maintenance engineers. The “intelligent diagnostic expert” is designed to provide maintenance engineers with smart diagnostic strategies so that they can accurately and quickly locate faulty units by taking reasonable testing steps.

Knowledge graph technology is an innovative tool that aims to mine entity relationships from vast, fragmented information and present them as structured semantic networks, which provide better methods for knowledge mining, representation, and management in the field of artificial intelligence [9,10,11]. With knowledge graph technology, fault knowledge can be extracted from vast, multi-source, heterogeneous fault documents [12]. By integrating the extracted information into a structured interconnected fault knowledge graph and applying reasoning technology, intelligent fault diagnosis strategies can be generated to guide maintenance engineers to locate the faulty unit quickly and accurately. As a result, an increasing number of scholars have started exploring the application of knowledge graph technology in the field of fault diagnosis. For example, Deng proposed a method to construct a fault diagnosis event logic knowledge graph for transmission systems using fault maintenance logs as the research object [13]. Similarly, Liu constructed knowledge graphs from maintenance records to describe the correlation between the fault components of transformers and improve fault diagnosis accuracy [14]. However, previous studies have focused mainly on constructing fault knowledge graphs, with a limited exploration of their practical applications. In particular, there is a significant gap in the literature regarding the construction and application of aircraft fault knowledge graphs. Against this background, this paper reviews the key technologies and application scenarios of knowledge graphs for aircraft fault diagnosis, with three contributions:

(a) Experts in the field of fault diagnosis were consulted to comprehensively sort out the elements of aircraft fault knowledge; identify the entity types, attributes, and relationship patterns involved in fault diagnosis; and construct the model of aircraft fault knowledge based on the practical needs of smart fault diagnosis for aircraft.
(b) A comprehensive fault knowledge extraction method based on deep learning and heuristic rules was used. Deep learning methods were primarily used to extract unstructured fault documents, while heuristic rules were employed to extract semi-structured data, tables, or normative documents with certain structural characteristics.
(c) A question-and-answer system based on aircraft fault knowledge graphs was developed, comprising question preprocessing, question analysis, graph retrieval, and answer generation modules.

## 2. Literature Review

### 2.1. Aircraft Fault Diagnosis

The existing methods of aircraft fault diagnosis can be divided into four categories, namely, model-based methods, signal-based methods, quantitative-knowledge-based methods, and qualitative-knowledge-based methods [15,16]. Table 1 sums up these methods from their principles, features, and application scopes.

From Table 1, we can see that qualitative-knowledge-based fault diagnosis methods are still the primary means of solving system-level fault localization problems, which is precisely the focus of this paper. One crucial point for such methods is that they require a reasonable model to describe fault knowledge [21]. Classical models such as fault tree models [22,23], Petri net models [24,25], rule models [26,27], and causality graphs [28] have been employed for constructing knowledge bases. However, these models have some drawbacks, such as the need for the analysis of potential equipment fault modes in advance, and artificial editing, making them inflexible and difficult to update dynamically. As a result, maintenance engineers’ experience and expertise in fault diagnosis cannot be shared systematically and timely. Nevertheless, fault diagnosis experience knowledge is invaluable, and if fully utilized, it can save human and material resources by eliminating the need for maintenance engineers to invest substantial efforts in analyzing the same faults repeatedly. Therefore, this paper proposes to use knowledge graph technology to mine fault knowledge from vast and diverse fault document data and then construct a structured and interconnected fault knowledge base.

### 2.2. Knowledge Graph

The concept of a knowledge graph was first introduced by Google in 2012, which involves using triplets of <entity–relation–entity> to form a semantic network. Entities are represented as nodes, and relations as edges, and their connections constitute the knowledge graph. The original intention of building a knowledge graph was to extract the relationships between entities from unstructured web information, integrate fragmented information into a coherent semantic network, and improve the quality and user experience of searches. At present, knowledge graphs have been widely used in the medical [29], education [30], finance [31], e-commerce [32], and safety [33,34] fields, among others, advancing AI from perceptual intelligence to cognitive intelligence [10,35]. For constructing a knowledge-domain graph, it involves two critical points: knowledge modeling and knowledge extraction.

Knowledge modeling is a critical step in designing domain applications by providing a logical schema layer for building knowledge graphs. The schema layer defines the types of entities, attributes, and relationships that may exist in the graph. Once the schema is established, instances of entities, attributes, and relationships can be extracted from unstructured documents automatically to build the knowledge-domain graph [36,37]. Typically, ontology modeling is used to construct the schema layer of a knowledge graph. Ontology offers a precisely defined conceptual layer for the knowledge domain [38]. For example, an ontology-based schema may contain “component-isPartOf-subsystem” while “receiver-isPartOf-radar” could be instances of this schema, i.e., a knowledge graph. Recently, researchers have more focused on ontology-based fault knowledge modeling to achieve fault knowledge extraction and sharing [39,40,41,42]. Therefore, ontology is well suited for describing a fault knowledge model, and knowledge graphs as instances of an ontology provide an ideal platform for acquiring and presenting fault knowledge.

Knowledge extraction mainly consists of two aspects: entity (attribute) recognition and relation recognition. The methods for entity and relation recognition can be primarily categorized into two types: rule-based methods based on linguistic heuristics, and statistical methods [43,44]. In the past, both rule-based and statistical methods required sufficient prior knowledge to design reasonable extraction rules or artificial features. However, with the rapid development of deep learning, it has become the mainstream approach for Knowledge extraction due to its advantages in handling massive amounts of data and its ability to automatically extract features from unlabeled data without a complex feature design procedure [43,44,45,46,47]. Despite this, rule-based methods still have their own advantages. For structured or semi-structured text, they offer a higher level of extraction precision. Many aircraft failure resources contain structured, semi-structured, and unstructured document data, where the structured and semi-structured data include Excel, XML, and standardized Word documents with specific structural characteristics, and the unstructured data include failure analysis articles and other Word documents. Hence, this paper proposes a comprehensive fault knowledge extraction method to deal with these various types of data. Specifically, the deep learning method is primarily used for the unstructured fault data, while heuristic rule methods are applied to structured and semi-structured data.

## 3. Methodology

### 3.1. Construction and Application Framework of Aircraft Fault Knowledge Graphs

**Figure 1:**
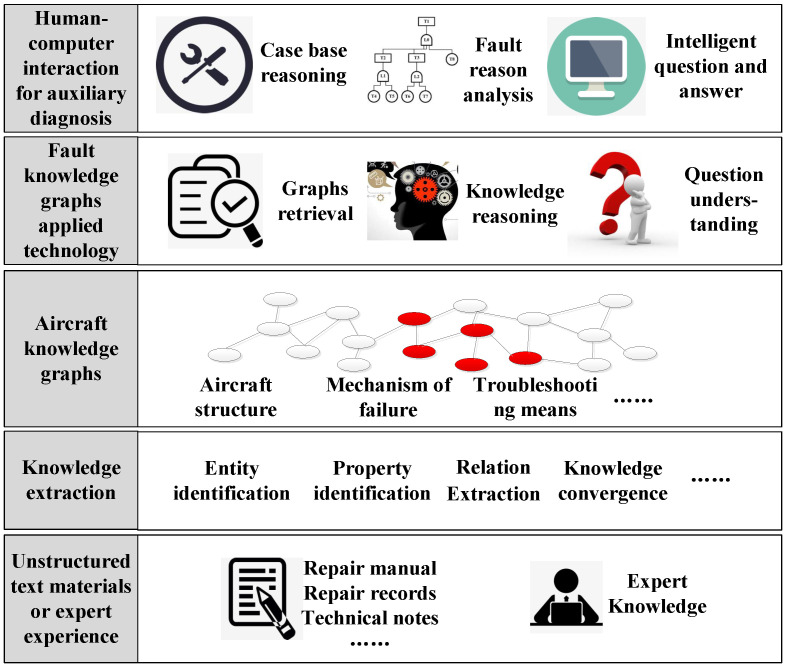
Construction and application framework of aircraft fault knowledge graphs.

Figure 1 illustrates the construction and application framework of fault knowledge graphs, which mainly consist of five layers. These are the fault data layer, knowledge extraction layer, knowledge graph layer, application technology layer, and human–computer interaction layer.

The fault data layer consists of a vast and varied amount of unstructured documentation related to faults accumulated over an aircraft’s lifecycle, such as FMECA documents, maintenance support textbooks, diagnosis records, and fault analysis reports.

The knowledge extraction layer involves identifying the entities, attributes, and relationships related to faults from the collected documents to build structured and interconnected fault knowledge graphs. This paper proposes a comprehensive fault knowledge extraction method that addresses various data types. Deep learning methods are primarily used for unstructured fault documents, while heuristic rule methods are applied to structured and semi-structured data.

The knowledge graph layer refers to the structured “large-scale” fault knowledge graphs extracted from massive documents. It is called “large-scale” due to the aircraft system’s complexity, with hundreds of thousands of components. When the failures, signal parameters, and composition relationships of each component are described, the number of nodes and edges in the graph can reach millions. To facilitate queries and updates of the large-scale graph, we need to use database management systems (DBMS), such as Graph DBMS and RDF Stores [48,49].

The application technology layer matches similar fault cases and infers possible fault causes using the fault knowledge graph. This layer can generate diagnosis strategies or answer maintenance engineers’ questions through semantic retrieval, knowledge reasoning, problem understanding, and other technologies.

The human–computer interaction layer refers to the visual interface facing directly towards the maintenance engineer. This interface includes the visual display of the fault knowledge graph, the visual interface of fault reasoning analysis, and interactive interfaces for intelligent question-and-answer sessions.

### 3.2. Design of Aircraft Fault Knowledge Graphs Schema

The schema layer, also known as the ontology layer, has a significant impact on the shape and quality of the fault knowledge graph, as well as its diagnostic efficiency. Therefore, when designing the schema for the aircraft fault knowledge graph, we adhered to several principles.

Firstly, the schema layer must be oriented towards fault diagnosis requirements. To achieve this, it is essential to fully understand the knowledge elements required for fault diagnosis by consulting with field experts and frontline aircraft maintenance personnel during the schema construction process. Through expert assessment, we aimed to create a fault-diagnosis-oriented schema that would be recognized by industry professionals.

Secondly, we acknowledge that despite the top-down approach generally used in schema design, continuous refinement is necessary based on feedback from the application and technical challenges encountered. For instance, during the initial stages of schema design, we defined various entity types, such as subsystems, assembly, components, and units at different levels. However, as we extracted knowledge, it became challenging to determine the precise level of a specific component from context alone. Consequently, we optimized the schema’s design and uniformly defined these entity types as a “unit”.

Thirdly, we strived to make the schema simple and effective. As the complexity of the schema increases, so does the effort required to extract entities and relationships from text due to extensive labor costs for corpus labeling. Therefore, while ensuring that the schema meets diagnostic business needs, it should be simplified as much as possible to avoid unacceptable manual labeling costs.

Finally, it is essential to consider both structured and unstructured documents when designing the schema since aircraft fault data comprise both formats. Structured data, such as Excel and standardized XML formats, contain rich information that can be directly converted into triples. Ideally, the schema should encompass all possible structured data information. On the other hand, unstructured data, mainly comprising Word and PDF documents, require deep learning and other techniques to extract knowledge. To simplify entity identification from unstructured documents, the schema assigns structured data information as attributes, reducing the number of entity types.

**Figure 2:**
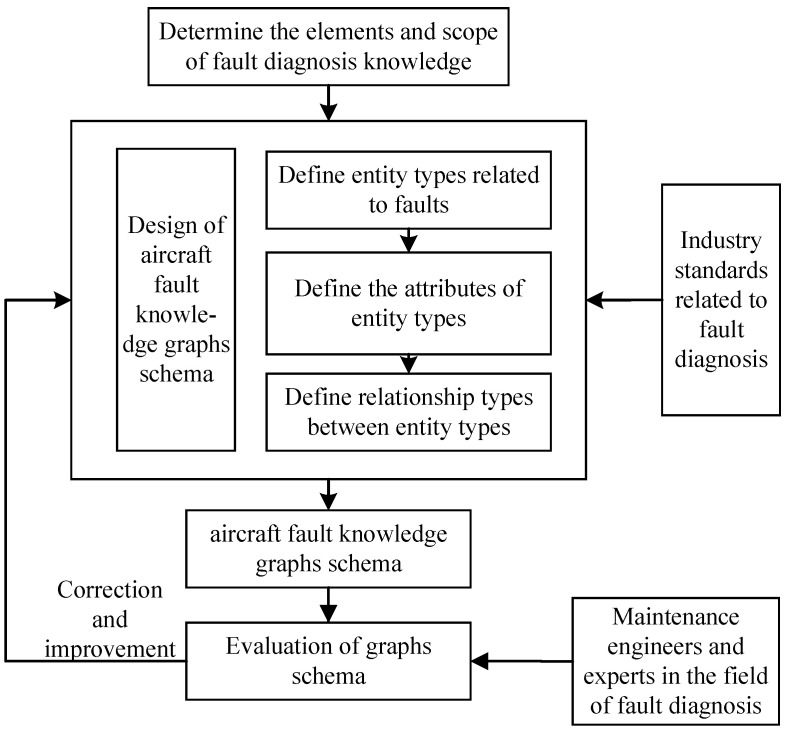
Evaluation iteration method for schema design.

Following the principles mentioned above, we adopted an “evaluation iteration method” to construct the fault knowledge graph schema, as shown in Figure 2. Relying on the evaluation of maintenance personnel and domain experts, the fault knowledge graph schema was continuously iterated and improved until a fault-diagnosis-oriented schema was built, which was unanimously recognized by experts in the field.

### 3.3. Fault Knowledge Extraction

Fault knowledge extraction is a process of identifying entity and relationship types from both structured and unstructured fault documents. To achieve this, the paper utilizes a comprehensive fault extraction method based on deep learning supplemented by heuristic rules. The deep learning method is primarily used for knowledge extraction on unstructured fault documents, while heuristic rule methods are applied to semi-structured data, tables, or standard documents with specific structural characteristics. The following section focuses on introducing the deep learning method for extracting information from unstructured documents.

#### 3.3.1. Entity Recognition

As previously discussed in this paper, the importance of deep learning in knowledge extraction cannot be overstated. One of the key benefits of deep learning is its ability to automatically discover features for classification or detection tasks. Recently, pre-trained language model embeddings, specifically BERT [50], have emerged as a promising approach for entity recognition [51]. There are various models for performing entity recognition based on BERT, some of which include BERT + Softmax, BERT + CRF, BERT + Span, and BERT + MRC [51,52,53]. Rather than treating entity recognition as a sequence labeling problem, BERT+MRC formulates it as a machine reading comprehension (MRC) task [52]. The method introduces label information by transforming the span extraction process into a question-and-answer format. However, Pan found that the BERT-MRC method does not fully leverage label information in span extraction. To address this issue, he proposed an enhanced language representation with label knowledge for span extraction, referred to as LEAR (Label-knowledge Enhanced Representation) [53]. This approach offers improvements over the BERT-MRC model by incorporating label information in a more effective manner.

**Figure 3:**
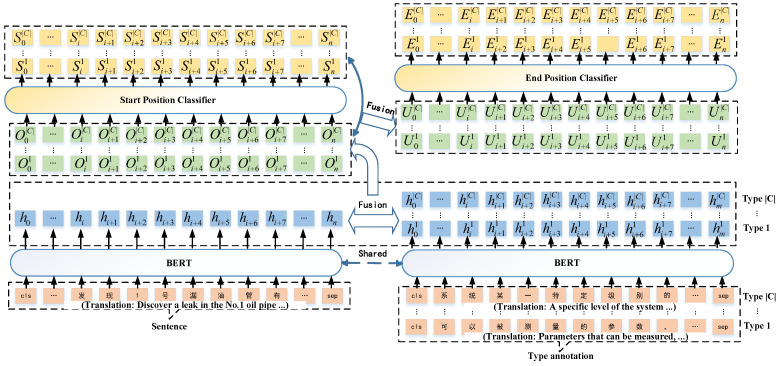
The framework of SP-LEAR.

The primary objective of span extraction is to identify the specific locations of entities within unstructured text. However, previous models have failed to account for the necessary prompt required to determine the end position given the starting point. In light of this limitation, we propose a novel solution, which involves integrating the precise starting position into the language representation using LEAR technology. This modified approach, which we refer to as SP-LEAR (Start Position and Label-knowledge Enhanced Representation), is illustrated in Figure 3.

The two key steps of SP-LEAR are as follows:

(1) Fusion of type annotations into sentences. Assume that *n* is the length of sentence, and *m* is the length of label annotation. After the process of the BERT network model, $h_{i}\in R^{d}$ is the embedding of the *i*th token of the sentence for any 1≤i≤n, and $h_{j}^{c}$ is the embedding of the *j*th token of the annotation of type *c* for any 1≤j≤m. To integrate the information from the label annotations into the sentences, an attention mechanism is employed. The attention score is computed using the following formula,
  (1) $$a\left(h_{i},h_{j}^{c}\right)=\frac{\exp\left(W^{q}h_{i}\right)\cdot \left(W^{k}h_{j}^{c}\right)}{\sum_{j}\exp\left(W^{q}h_{i}\right)\cdot \left(W^{k}h_{j}^{c}\right)}$$
  where $W^{q},W^{k}\in R^{d\times d}$ are learnable parameters of the fully connected layers. Then, the fusion features are obtained via
  (2) $$O_{i}^{c}=\tanh\left(W^{v}\left(W^{q}h_{i}+a\left(h_{i},h_{j}^{c}\right)W^{k}h_{j}^{c}\right)+b\right)$$
  where tanh(·) is the hyperbolic tangent function, and $W^{v}\in R^{d\times d}$ and $b\in R^{d}$ are learnable parameters of the fully connected layers.
(2) Fusion of start position into features. The fused features are used as input to the start position classifier in order to output the probability of the *i*th token as the starting position of a span of the category *c*, denoted as $S_{i}^{c}$. The new features $U_{i}^{c}$ are created through concatenating the fusion features and start position probability using the following formula
  (3) $$U_{i}^{c}=\tanh\left(M\cdot \mathrm{concat}\left(O_{i}^{c},S_{i}^{c}\right)\right)$$
  where $M\in R^{d\times d}$ is learnable parameters of the fully connected layers.

To enhance the end position classification, the newly extracted span features are used as input. This allows for obtaining the probability of the *i*th token being the ending position of a span belonging to category *c*.

The loss function of the model is defined to be $L=L_{s}+L_{e}$, where $L_{s}$ and $L_{e}$ are, respectively, start loss function and end loss function, and the two values are calculated by cross entropy. The nearest matching principle [39], which matches a starting position of category *c* with its nearest next end position, is used for decoding the span.

#### 3.3.2. Relation Extraction

The relationship extraction method integrates both rule-based and model-based approaches. The rule-based method draws on our knowledge of the fault data and logical connections, requiring the manual creation of extraction rules, regular expressions, and templates. For example, when dealing with equipment technical documentation, the composition relationship between fault units can be directly extracted through the directory relationship of each composition unit. Additionally, if a sentence exhibits keywords such as “cause” or “lead to” between two fault entities, it implies a causal relationship between them. Such rules allow for precise extraction of relationships in a straightforward and efficient manner.

**Figure 4:**
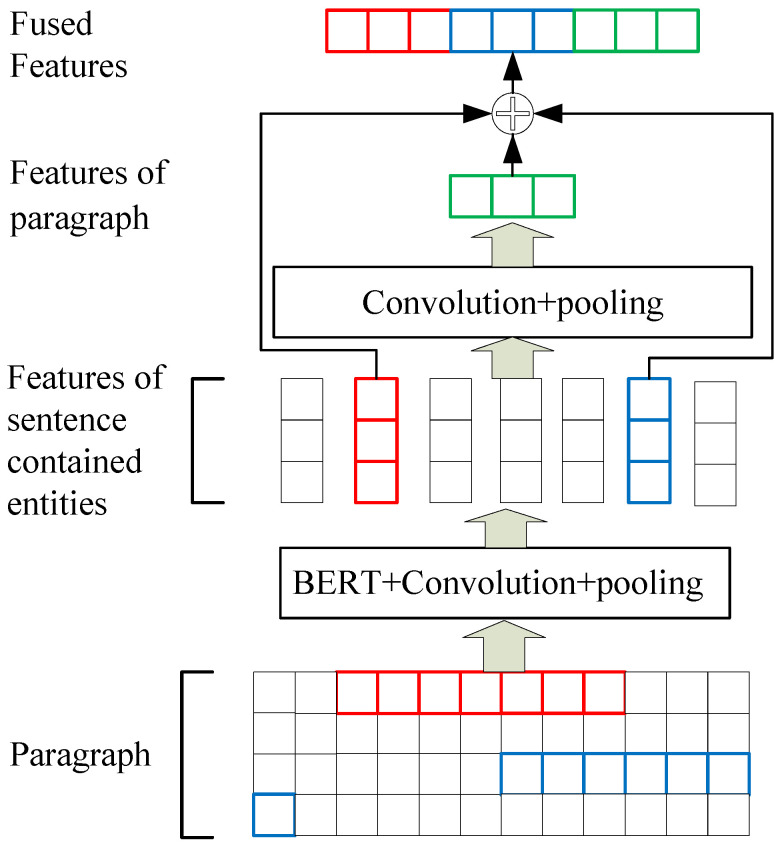
The feature extraction for relationship classification.

The deep learning method is also employed to extract relationships. This method transforms relationship extraction into a binary classification task, where the model predicts whether a predefined relationship exists between two entities within a particular paragraph. The classifier features were obtained through a multi-step process illustrated in Figure 4. Firstly, the model locates sentences containing entity pairs and extracts their features using the “BERT + Convolution + Pooling” model. The second step involves extracting paragraph-level features through the “Convolution + Pooling” model, which are then concatenated with the sentence features that contain entity pairs to serve as input for the classifier model. To illustrate, let us consider the following example: “…the alarm system continuously reflects that the right engine speed is too high during the descent phase. The reason is that the oil level sensor control component fails, …”. Both of these sentences comprise a fault entity. In this case, the features of the two sentences, along with the paragraph in which they appear, are concatenated, and a classification model is used to determine whether or not a causal relationship exists between the two fault entities.

### 3.4. Question-Answering System Based on Fault Knowledge Graph

#### 3.4.1. The Development of Question-Answering System

**Figure 5:**
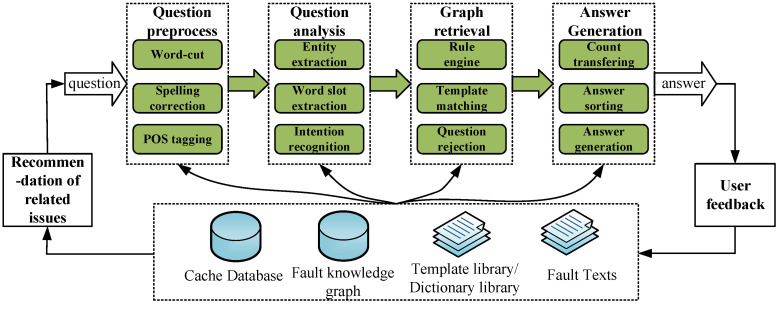
The framework of question-answering system.

To assist maintenance personnel in effectively identifying the root cause of faults, an intelligent question-answering system was developed using the fault knowledge graph. The system consists of four major modules: a question preprocessing module, a question analysis module, a graph retrieval module, and an answer generation module, as shown in Figure 5.

The question preprocessing module is responsible for performing essential tasks such as spelling correction, word segmentation, and part-of-speech tagging on input queries to extract lower-level, granular question information.

Additionally, the question analysis module consists of two critical components: a word slot extraction unit and an intention recognition unit, which are designed to extract word slots and recognize the intention of pre-processed questions, respectively. By utilizing precise or fuzzy matching templates, this module can effectively analyze relevant slot data from the question, including entity slots, schema slots, attribute slots, condition slots, and more, that have been predefined by intention template. For instance, consider the query “How many components are there in the braking system?” The resulting extracted slots would be the entity slot “braking system”, schema slot “unit”, and condition slot ”how many”.

Once the intention template has been identified, the graph retrieval module retrieves pertinent fault information from the knowledge graph module. This is achieved by translating the intent template data into AQL query statements using rule heuristics and template matching, which connects to ArangoDB and retrieves relevant entity and relationship names, attribute values, and other information from the database. Additionally, this module also searches for relevant documents based on the retrieved entities, ensuring complete knowledge traceability.

Lastly, the answer generation module is responsible for converting and merging the database results based on answer templates, sorting the answers, and returning an answer set along with any related documents.

#### 3.4.2. The Deployment of Question-Answering System

**Figure 6:**
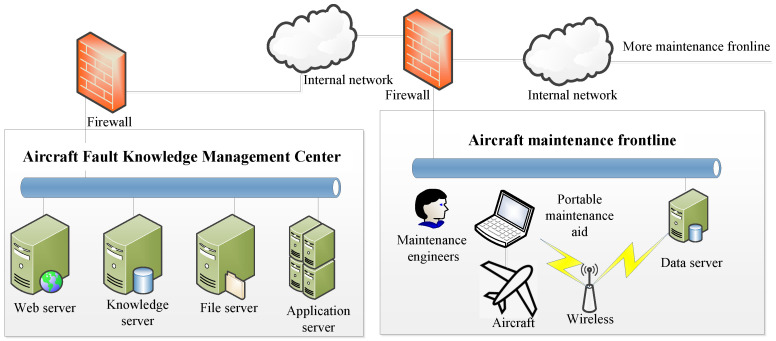
The deployment of question-answering system.

Figure 6 illustrates the deployment of our question-answering system, which is situated in an aircraft fault knowledge management center. Using portable maintenance aid, maintenance engineers at the maintenance forefront can gain access to the knowledge graph and intelligent question-answering system, enabling them to swiftly retrieve the information they require by simply posing questions. This capability enables them to locate faulty units within a short timeframe. After completing the aircraft fault diagnosis and maintenance work, the maintenance engineers document relevant information such as fault phenomena, fault cause analysis, and fault elimination experiences online. The documented data are then integrated into the fault knowledge management center. Through our model, fault knowledge is extracted from this information, thereby facilitating regular updates of the knowledge graph and achieving the objective of sharing fault diagnosis experience.

## 4. Results

### 4.1. The Schema of Fault Knowledge Graph

**Figure 7:**
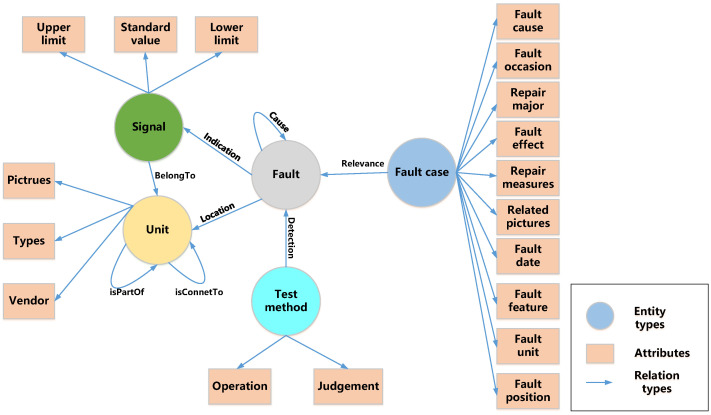
The schema of fault knowledge graph.

Based on the evaluation iteration method, we performed several optimization iterations to establish the schema presented in Figure 7. Table 2 and Table 3 illustrate the definitions of the entity types and their relationships, which include “unit”, “signal”, “fault”, “fault case”, and “test method” entities. It is worth noting that the schema does not differentiate between fault phenomena, modes, and causes as separate entities. Instead, it considers them collectively as “fault”. This decision was based on the observation that these three types of entities are expressed similarly in unstructured text. For instance, the fault phenomenon “right engine speed is too high” may correspond to the fault cause “right engine FRV cannot be opened”. In this case, machine learning algorithms may not reliably differentiate between these entities based on contextual features alone. Additionally, distinguishing between these entities adds little value to fault diagnosis, since establishing a “cause” relationship between faults A and B suffices to infer that A is the fault cause of B. Furthermore, faults can form propagation chains where one fault triggers another, ultimately leading to yet another. In such cases, identifying the component in the propagation chain where the fault occurred becomes necessary. Defining “fault cause” as a distinct entity would complicate the description of fault propagation chains. Thus, for simplicity, “fault” and its “cause” relationship are defined in the schema.

**Figure 8:**
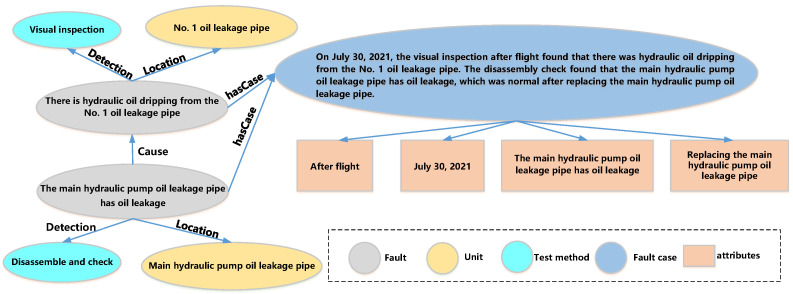
An example of local knowledge graph under defined schema.

As shown in Figure 8, an example of a local knowledge graph under the defined schema illustrates how the elements in the graph shall be extracted from fault documents using specified methods.

### 4.2. Fault Knowledge Extraction

We labeled 4305 entities from a total of 1000 paragraphs, where 700 paragraphs were allocated for the training set and 300 paragraphs for the testing set. In order to accurately evaluate the performance of entity recognition methods, this study uses precision (*P*), recall (*R*), and F1-score ($F_{1}$) to assess model performance. The calculation methods for each evaluation metric are as follows.

(4) $$P=T_{P}/\left(T_{P}+F_{P}\right)$$

(5) $$R=T_{P}/\left(T_{P}+F_{N}\right)$$

(6) $$F_{1}=\left(2\times P\times R\right)/\left(P+R\right)$$

where $T_{P}$ represents the number of true positive samples of predicted fault entities, $F_{P}$ represents the number of false positive samples of predicted fault entities, and $F_{N}$ represents the number of false negative samples of predicted fault entities.

We conducted experiments on different models using this dataset, and we present a comparison of the results in Table 4. The best-performing model’s results are highlighted in bold. It is evident that the direct classification of BERT embeddings using Softmax performs poorly because it fails to consider the character dependencies. The “BERT + CRF“ entity recognition model, which uses a CRF decoder to determine each token’s “BIO” label, outperforms Softmax but falls short of “BERT + span”. LEAR is an improvement over “BERT + span” as it includes label information when generating features, resulting in higher recall. Compared to LEAR, our method incorporates the starting position into features when classifying the ending position. The results indicate that although SP-LEAR has lower recall when compared to LEAR, accuracy improved significantly, producing a higher $F_{1}$ value.

The performance of SP-LEAR in different entity recognition conditions is shown in Table 5. The results indicate that the extraction of “Unit” and “Signal” entities performed much better than that of the “Fault” and “Test method” entities. This could be attributed to the fact that “Unit” and “Signal” are nouns with clearly defined boundaries and shorter length, while “Fault” and “Test method” are short sentences that contain predicates with relatively vague boundaries. Therefore, further research is needed to improve the effectiveness of extracting “Fault” and “Test method” entities.

**Figure 9:**
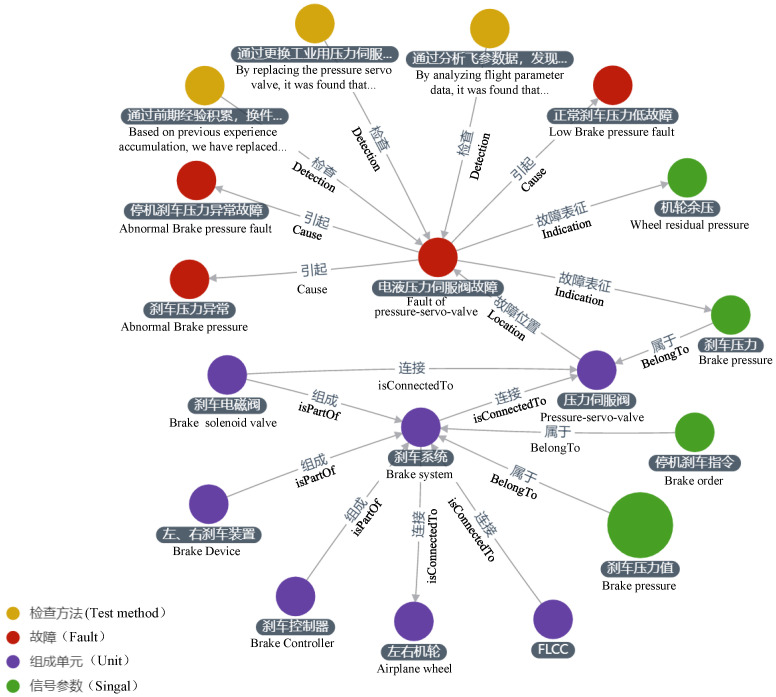
Visual display of fault knowledge graph.

With the technology of fault knowledge extraction, a fault knowledge graph for a certain type of aircraft is constructed, and Figure 9 shows the local fault knowledge graph of an aircraft braking system, including important information such as the composition structure of the system, signal parameters, related faults, and fault detection methods. This fault knowledge graph provides crucial knowledge support for fault diagnosis. Additionally, to facilitate query and updates of the large-scale graph, we need to use database management systems to store the aircraft fault knowledge graph. ArangoDB and Neo4j are widely used graph databases. However, compared to Neo4j, ArangoDB is an open-source-distributed native multi-model database with advantageous features, such as multi-model support, scalability, and high performance. One noteworthy feature of ArangoDB is its unified query language, AQL, which is similar to SQL. This allows for rapid graphical queries that return nodes, edges, and their attributes. These characteristics make ArangoDB particularly well suited for storing triplets in fault knowledge graphs.

### 4.3. Question-Answering System

**Figure 10:**
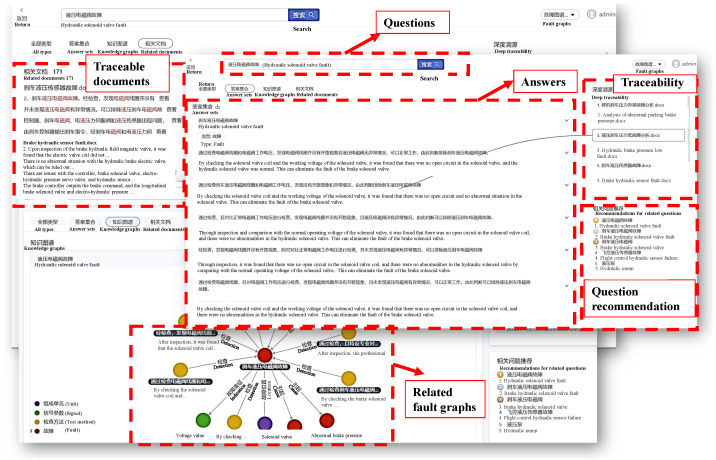
The application of question-answering system.

Figure 10 provides a depiction of the interface and an application example of the intelligent question-answering system. The example reveals how the system can assist maintenance engineers encountering abnormal brake pressure failures. An engineer simply inputs the query “How to handle abnormal brake pressure?” into the system, which then identifies various reasons behind the occurrence. Each reason is further accompanied by relevant detection methods. By using these prompts, the maintenance engineer can quickly identify the root cause of the problem. Moreover, the system comes equipped with a knowledge tracing function that allows users to trace the origin of each answer back to its source document. This feature proves especially useful for reference purposes, as maintenance engineers may need to delve further into provided documents to better understand a given issue. Finally, through the use of fuzzy matching, the system is capable of providing similar fault cases or problems that arise in related contexts, enabling maintenance engineers to consider previously solved cases when troubleshooting.

To evaluate the effectiveness of the fault knowledge question-answering system, we enlisted the participation of twenty grassroots maintenance engineers to conduct a testing process. Each engineer was instructed to provide a set of twenty frequently asked questions which commonly arise during fault diagnosis. Subsequently, the engineers evaluated the system’s answers and marked them as satisfactory or unsatisfactory according to their level of adequacy. The results of this assessment revealed that the knowledge question-and-answer system achieved a high level of satisfaction, with 86.5% of the engineers rating it positively.

## 5. Conclusions and Discussion

This paper reviews the technologies as well as the application scenarios of knowledge graphs for aircraft fault diagnosis. Firstly, we identified knowledge elements based on the requirements for aircraft fault diagnosis and constructed a schema layer for the aircraft fault knowledge graph. Secondly, we conducted a preliminary investigation into the extraction of fault knowledge from structured and unstructured data. Using this approach, we constructed the fault knowledge graph for a specific type of aircraft and accomplished the storage and visualization display of the graph. Finally, we developed a question-answering system based on the constructed graph that can accurately answer the questions posed by maintenance engineers and trace the documents where the answers were found.

Our exploration shows that knowledge graph technology can fully exploit structured and unstructured fault data and facilitate the integration and correlation of resources into a unified fault knowledge base. By providing a means for sharing and utilizing aircraft fault knowledge, knowledge graphs offer significant implications for improving diagnostic efficiency and accuracy. Overall, our results suggest that knowledge graphs offer a promising approach to aircraft fault knowledge management. However, there are limitations to this study that we need to explore in the future.

The current exploration applies traditional knowledge graphs to the field of fault diagnosis. However, such graphs mainly establish static knowledge bases that focus on nouns, describing “what is” and what “relationships exist”. Conversely, aircraft fault knowledge involves some dynamic predicate events requiring dynamic knowledge, such as “why and how”, to be described. For example, the fault phenomenon “right engine speed too high during the descent phase” is an event rather than a noun. Empirical evidence showed that treating the event solely as a static entity could lead to various issues. On the one hand, some common entity recognition methods may have low recognition accuracy and recall for events described by short sentences with fuzzy boundaries. On the other, finer-grained knowledge embedded within the event may be overlooked entirely. For this point, we will attempt to integrate event models into the built knowledge graph to provide better descriptions of fault logic in the next work.

The built fault knowledge graph in this study is a text knowledge base in nature. However, our collected fault data in practice contain numerous images, such as system structural composition diagrams and abnormal pictures of faulty components, and there may be videos containing information, such as part disassembly and installation. This information cannot be portrayed by our current knowledge graph. Therefore, for this point, we will develop a multimodal knowledge graph that contains images, videos, and other forms of knowledge to enrich the fault knowledge base.

The question-answering system developed in this study serves as a knowledge-retrieval tool, but it has limitations in achieving natural and effective multi-round conversations. In order to enhance the system’s functionality, we propose incorporating knowledge graph technology with ChatGPT technology to develop an advanced interactive fault diagnosis system. ChatGPT technology exhibits robust information integration and dialogue capabilities, making it well suited for application in the field of aircraft fault diagnosis. However, ChatGPT does have some drawbacks. Due to its opaque nature and lack of traceable knowledge, there is a risk that it may generate erroneous or misleading information, which could pose practical problems for maintenance engineers. To address this issue, we plan to integrate knowledge graph technology into the diagnostic system, thereby creating a more efficient and reliable interactive diagnostic assistant. Specifically, we will retrieve relevant knowledge elements from the fault knowledge graph and use them to prompt a natural language generation model to produce trustworthy and accurate responses. By combining these two technologies, we aim to create a system that not only provides efficient fault diagnosis but also ensures the reliability and traceability of the information generated.

## Figures and Tables

**Table 1:** Analysis of different fault diagnosis methods.

Methods	Principle	Features	Application Scopes
Model-based fault diagnosis	It constructs an accurate physical model to describe the dynamic changes in the system, and then diagnoses faults by monitoring the consistency between the actual measurement output of the system and the predicted output of the model.	Its accuracy is very high, but it requires the establishment of accurate and quantitative physical models.	Its application is generally limited to local controllers of aircraft [17].
Signal-based fault diagnosis	It diagnoses faults by analyzing and processing monitored signals in the time or frequency domain.	It does not need to establish physical models and only analyzes and processes the signals of sensors.	It is typically used to detect early faults or identify fault types on key components that are being monitored in real time [18]. However, it is not suitable for locating faulty units.
Quantitative-knowledge-based fault diagnosis	It treats fault diagnosis as a pattern recognition problem. It involves extracting features from the fault-related dataset and training a model through historical data that can identify different fault states or modes.	It does not need to establish physical models, but it requires a large amount of historical fault data.	It is typically used to detect early faults or identify fault types on key components that have accumulated a large amount of historical data [19,20]. However, it is not suitable for locating faulty units.
Qualitative-knowledge-based fault diagnosis	It constructs a qualitative model to describe prior knowledge related to faults and then diagnoses faults through searching, matching, reasoning, and other techniques.	It does not require the construction of accurate physical models or extensive historical fault data but does require building a complete fault knowledge base.	It is frequently used to locate faulty components in complex systems.

**Table 2:** Definition of entity type.

Entity Type	Connotation
Unit	An element of the system at a specific level (subsystem, assembly, component, etc.). A unit can be composed of several subunits, or it can be a subunit of a larger unit. Its attributes include types, vendor, and picture.
Signal	Parameters that can be measured. Many faults may be characterized as abnormal signals. Its attributes include standard value, upper limit, and lower limit.
Fault	Description of all abnormal events, such as abnormal function, parameter deviation, mechanical damage. It includes fault phenomenon, fault mode, and fault cause.
Fault case	Specific occurrences of a fault. A fault may occur multiple times at different times and places, and each occurrence is considered a separate fault case. It includes attributes such as fault time, fault location, and fault effect.
Test method	The method used to detect a fault, which guides maintenance engineers to quickly locate the fault unit. It has two attributes: operation and judgement.

**Table 3:** Definition of relation type.

Relation Type	Connotation	Head Entity	Tail Entity
Cause	Indicates causality between faults.	Fault	Fault
Detection	Indicates that a fault can be detected using a test method.	Fault	Test method
isPartOf	Indicates that a unit is part of another unit.	Unit	Unit
isConnectTo	Indicates physical contact or signal input/output relationship between units.	Unit	Unit
BelongTo	Indicates that a signal belongs to a unit.	Signal	Unit
Location	Indicates that a fault occurs at a unit.	Fault	Unit
Indication	Indicates that a fault can be indicated by a signal.	Fault	Signal
Relevance	Indicates that a fault case is relevant to a fault.	Fault case	Fault

**Table 4:** Results comparison of different models.

Model	*P*	*R*	$F_{1}$
BERT + Softmax	0.283	0.430	0.341
BERT + CRF	0.757	0.797	0.778
BERT + SPAN	0.796	0.799	0.797
LEAR	0.797	**0.833**	0.814
SP-LEAR	**0.813**	0.827	**0.820**

The best-performing model’s results are highlighted in bold.

**Table 5:** The performance of SP-LEAR in different entity recognition conditions.

Entity Category	*P*	*R*	F${}_{1}$
Unit	0.908	0.908	0.908
Signal	0.833	0.894	0.863
Fault	0.820	0.790	0.804
Test method	0.780	0.832	0.805

## Data Availability

Data is unavailable due to privacy.
